# Assessment of knowledge and education relating to asthma during pregnancy among women of childbearing age

**DOI:** 10.1186/s40733-017-0038-x

**Published:** 2018-01-19

**Authors:** Mohammed O. Al Ghobain, Mohammed AlNemer, Mohammad khan

**Affiliations:** 1 Department of Medicine, King Abdullah International Medical Research Center, College of Medicine, King Saud bin Abdulaziz University for Health Sciences, P.O. Box 90068, Riyadh, 11321 Saudi Arabia; 2 Department of Medicine, King Abdulaziz Medical City, Riyadh, Saudi Arabia

## Abstract

**Background:**

Misconceptions about medications’ safety can lead pregnant women with asthma to stop their medications, resulting in asthma-related neonatal morbidity and mortality. Our aim was to assess the level of pregnancy-related asthma knowledge and education about asthma medications’ safety, among women of childbearing age with a history of bronchial asthma.

**Methods:**

A cross-sectional survey of convenience sample of outpatient clinic attendees of Pulmonary, Family Medicine and Obstetrics & Gynecology among women of childbearing age with history of asthma at King Abdulaziz Medical City, Riyadh, Saudi Arabia. Participants (*n* = 171) completed a questionnaire to determine levels of education and knowledge, as well as attitudes and practice relating to asthma treatment.

**Results:**

Among participants, 77.1% were pregnant at the time of the survey, 77.8% had used asthma medications during current or previous pregnancy, 70.8% of all respondents who ever been pregnant believed in the safety of asthma medications during pregnancy, 49.1% had received education about asthma, and 46.8% had been educated about the safety of asthma medications during pregnancy. Responses indicated that 46.8% had stopped (or expressed the desire to stop) asthma medications during pregnancy, and 48% believed asthma medications would harm them and their babies more than asthma itself, but 92.4% expressed that they would be willing to use asthma medications during pregnancy if their safety was confirmed by a physician. Education level and employment status were both associated with an increased likelihood of having received asthma education (*p* values <0.001 and <0.001 respectively), and with awareness of the safety of the medications during pregnancy (*p* values <0.001 and <0.003 respectively).

**Conclusion:**

Further efforts is to be taken to develop a program where female asthmatic patients are taught about asthma and its medications’ safety during pregnancy.

## Background

Asthma is a heterogeneous disease characterized by chronic airway inflammation and is defined by a history of respiratory symptoms together with variable expiratory airflow limitation confirmed by pulmonary-function tests, and its management is accomplished through the use of β2-adrenoceptor agonists and inhaled corticosteroids [1].

The results of a national household survey in Saudi Arabia in 2013 that estimated the burden of chronic medical conditions among individuals ≥15 years old indicated a self-reported prevalence of asthma of 4.05% [2]. According to an update published by the Saudi Initiative for Asthma (SINA) in 2016, the overall prevalence of asthma in Saudi children ranges from 8% to 25% [3]. Although no information is available on the prevalence of the disease among pregnant women in Saudi Arabia, or the levels of use of medications to control it in this population, asthma is estimated to affect 8–10% of women of child-bearing age in the U.S.A. [4] Accordingly, it is now thought that asthma might be the most common serious complication that is underdiagnosed during pregnancy [5].

A number of physiological changes occur in the respiratory system during pregnancy, including increases in tidal volume, resting minute ventilation and airway conductance, and decreases in residual volume and total pulmonary resistance [6]. The respiratory effects of uncontrolled asthma in pregnant women may counteract these adaptations, potentially increasing the incidence of pre-eclampsia, preterm labor, fetal growth restrictions, perinatal morbidity, spontaneous abortion, low birthweight, and cesarean delivery [7].

To avoid asthma-related complications, goal-oriented management should be carried out to encourage patient compliance with approved treatment regimens, through education about the safety of medications. Non-compliance in pregnant women with asthma is mostly caused by concern that the fetus will be affected by the treatment regimen arising from a lack of education about both the risks associated with the disease and the safety of the medications such a fear is a claim often exaggerated with no solid grounds. As investigated by National Institutes of Health, National Heart, Lung and Blood Institute asthma medications are entirely safe during pregnancy and free from risks and adverse events [8] In an Australian study addressing levels of asthma self-management skills and knowledge among 211 pregnant women with asthma, 42% had poor knowledge relating to their asthma medication, 40% reported non-adherence to their inhaled corticosteroids, and only 15% had a written action plan [9]. In another study in Alabama, US involving 501 women of childbearing age who had asthma, 82% reported being afraid to use inhaled corticosteroids, as they believed the drugs might cause congenital malformations, low birthweight, or even fetal death. In that study, 39% of the participants had discontinued or reduced their asthma medication during pregnancy, and a third of these non-compliant participants did so without consulting a physician [10].

In the present study, our aim was to assess the level of asthma knowledge and education about relevant medications’ safety and compliance during pregnancy among women of childbearing age with asthma in Saudi Arabia which has never been investigated to the best of our knowledge, and to identify the areas where knowledge is lacking.

## Methods

This study used cross-sectional convenience sampling of women of childbearing age (15–45 years old) with a history of bronchial asthma, attending pulmonary, obstetrics and gynecology, and family medicine clinics at King Abdulaziz Medical City, Ministry of National Guard Health Affairs, Riyadh, Saudi Arabia. Participants were recruited by first identifying all patients coming to pulmonary, Family Medicine and Obstetrics & Gynecology clinics and visually examining their files for a physician’s diagnosis of asthma in various medical records. Then, the identified patients were called from the waiting room and invited to participate in the study, the purpose of which was explained. If they agreed the informed consent taken from the participants (women in childbearing age) and then were taken to a separate room to fill out the survey and answer questions.

A self-administered questionnaire was constructed for the study, and was expanded on the basis of a literature review of the topic, and input from four expert consultants from the departments of Pulmonology and Obstetrics and Gynecology, to ensure thorough coverage of the points addressed in the survey. A pilot study was carried out to assess clarity and to gain feedback, which was incorporated into the finalized questionnaire.

Demographic variables were summarized and reported across the study cohort using descriptive statistics. Because the outcomes of interest were dichotomous, simple binary logistic regression analyses were conducted. Differences in distributions of categorical variables were determined by Chi-square tests. Associations between exposures and outcomes were considered statistically significant for *p* ≤ 0.05. Analyses were conducted using SPSS (V.22).

## Results

A total of 171 patients were included, 131 (77%) of whom were pregnant at the time of the study and 40 patients had been pregnant before (23%) (Table 1). All but one of the patients were currently prescribed asthma medication, including short-acting β-agonists (*n* = 149), a combination of inhaled corticosteroids and a long-acting β-agonist (*n* = 92), inhaled corticosteroids alone (*n* = 11), daily prednisone (*n* = 5) and the leukotriene-receptor antagonist (*n* = 1), (Table 1).

**Table 1:** Demographic and Clinical Characteristics of the Study Cohort

Demographic Characteristic		
Age (years)	*Mean ± SE*	32 ± 0.48
Asthma chronicity (years)	*Mean ± SE*	11 ± 0.64
Months of gestation	*Median*	7.00
Current pregnancy		(131) 77.2%
Asthma symptoms during any pregnancy		(136) 79.5%
Educational level	Below university	(89) 52%
	University and above	(82) 48%
In employment		(57) 33.3%
Asthma medications:		
Short-acting β-agonist		(149) 87.1%
Combination therapy (inhaled corticosteroids/long-acting β-agonist)		(92) 53.8%
Leukotriene-receptor antagonist		(1) 0.6%
Inhaled corticosteroids alone		(11) 6.4%
Theophylline		(0)
Prednisone		(5) 2.9%
Ant-IgE		(0)
No prescribed treatment		(1) 0.6%

With regard to using asthma medications during pregnancy, 133 (77.8%) of the study participants have or had used asthma medications during pregnancy, and 121 (70.8%) indicated that they believed that such use was safe (Table 2). Only 84 patients (49.1%) had received education about asthma, and only 77 (45%) had been educated about the safety of asthma medications during pregnancy. A total of 80 (46.8%) had stopped (or expressed the desire to stop) their asthma medications during pregnancy, 89 (52%) believed asthma medication would harm them and their babies more than asthma itself, and 158 (92.4%) expressed the willingness to use asthma medications if a physician had consulted them and indicated that the use of such medications during pregnancy was safe (Table 2).

**Table 2:** The level of asthma education and knowledge about medications’ safety and compliance during pregnancy among women of childbearing age with asthma in Saudi Arabia

Have used asthma medications during pregnancy	(133) 77.8%
Believe that asthma medications are safe to use during pregnancy	(121) 70.8%
Have received education about asthma	(84) 49.1%
Have received education about the safety of asthma medications	(77) 45%
Have stopped (or expressed the desire to stop) asthma medications during pregnancy	(80) 46.8%
Believe that asthma will harm patient and her baby more than asthma medications	(89) 52%
Willing to use medication if a physician provides confirmation of safety in pregnancy	(158) 92.4%

In a bivariate analysis of educational level with knowledge and perception, patients who had an education to university level or above were more likely to be educated about asthma (*p* < 0.001) and to be aware of the safety of asthma medications during pregnancy (*p* < 0.001) than patients educated to a lower level. The level of education was not associated with significant differences in any other parameters (Table 3). A similar relationship was observed between patients who were in employment and those who were not (Table 3).

**Table 3:** Bivariate analysis of educational and employment level with knowledge and perception about medications’ safety and compliance during pregnancy among women of childbearing age with asthma in Saudi Arabia

	Education level			Employment		
	Below university (89)	University and above (82)	*P* value	Employed (57)	Not employed (114)	*P* value
Patients used asthma medications during pregnancy	(70) 78.7%	(63) 76.8%	0.775	(45) 78.9%	(88) 77.2%	0.795
Patients think asthma medications are safe during pregnancy	(62) 69.7%	(59) 72%	0.742	(40) 70.2%	(81) 71.1%	0.905
Patients received education about asthma	(28) 31.5%	(56) 68.3%	<0.001	(40) 70.2%	(44) 38.6%	<0.001
Patients educated about the safety of asthma medications	(31) 34.8%	(49) 59.8%	<0.001	(36) 63.2%	(44) 38.6%	0.003
Patients stopped (or the will to) of Asthma medications during pregnancy	(47) 52.8%	(44) 53.7%	0.911	(24) 42.1%	(56) 49.1%	0.386
Patients believe that asthma will harm patient and her baby more than asthma medications	(39) 43.8%	(50) 61%	0.0249	(38) 66.7%	(51) 44.7%	0.007
Patients will use medications if a Physician consulted them about medications safety	(85) 95.5%	(73) 89%	0.110	(50) 87.7%	(108) 94.7%	0.128

No significant difference was observed in the use of asthma medications during pregnancy between women who were educated about asthma and those who were not (*p* = 0.493). However, patients who had received education about asthma during pregnancy were significantly more likely to report that they believed in the safety of asthma medication during pregnancy than patients who had not received such education (*p* = 0.0004).

## Discussion

Our results provide insight into the level of asthma education, and of knowledge relating to medications’ safety and compliance during pregnancy, among asthmatic women of childbearing age. Almost half of the participants had never received education about asthma and had never been told about the safety of using the asthma medications during pregnancy. However, those who had used asthma medications during pregnancy were in the majority, those who reported believing in the safety of those medications. Nevertheless, almost half of the participants had stopped (or expressed the desire to stop) taking asthma medications during pregnancy. Patients with high levels of education, and those in employment, were likely to have received asthma education, and to be aware of the safety of the medications. Receipt of asthma education was associated with belief in the safety of the medications during pregnancy.

Maternal asthma has been shown to be associated with the risk of low birthweight, small-for-gestational-age status, preterm delivery and pre-eclampsia. The association with these complications is reduced to non-significant levels by active asthma management [11]. Asthma education during pregnancy is an important part of disease management, because poor compliance with medications can have serious and potentially fatal consequences for both mothers and babies. These consequences are easily avoidable and reversible with education that results in treatment compliance. Unfortunately, asthma education during pregnancy has not yet been widely adopted into routine practice. Furthermore, asthma in pregnancy has not been widely investigated. Our results demonstrate that there is considerable scope to provide appropriate education to Saudi women with asthma.

Non-adherence to asthma medications is a common problem during pregnancy, mainly related to misconceptions regarding treatment safety [10]. For example, in a study involving 501 women of childbearing age with asthma, 82% of those who used inhaled corticosteroids were concerned about their effects on the fetus, as well as the consequences of discontinuing the medication on their own health. Among these women, 39% had discontinued the medication while pregnant, without consultation with their physicians [10]. Because of the adverse effects of uncontrolled asthma, The Global Initiative for Asthma (GINA) guidelines state that pregnant patients with asthma should be advised that poor control and exacerbation of the condition provide a much greater risk to the fetus than currently available treatments, and the therapeutic approach to asthma should be the same in pregnant and non-pregnant patients [1, 12].

Our findings demonstrate that patients who have received education relating to asthma in pregnancy are more likely to believe in the safety of appropriate medications than patients who have not received such education. This finding reinforces the importance of education relating to asthma in pregnancy, and should encourage health-care workers to implement educational programs for women of childbearing age. This result is in line with findings from Australia, in which 42% of 211 pregnant women with asthma had poor knowledge relating to medications, and 40% reported non-adherence to inhaled corticosteroids [9]. That study found that after receipt of asthma education, knowledge about medication safety improved to 95%, and non-adherence decreased to 21% [9]. In a US study of pregnant women with asthma, medication use was found to decrease significantly (*p* ≤ 0.0005) from 5 to 13 weeks of pregnancy [13]. During the first trimester, there was a 23% decline in inhaled corticosteroid prescriptions, a 13% decline in short-acting β-agonist prescriptions, and a 54% decline in rescue corticosteroid prescriptions [13]. Overall, the women’s perception about the disease, and their education relating to asthma medications, were not satisfactory to achieve adherence to medications preventing poor feto-maternal outcomes, but women with high levels of formal education, as well as those in employment, were likely to have received asthma education, and to be aware of the safety of the medications [13].

Our study was, however, limited to patients attending a single center, so care should be taken when generalizing the results, although studies at different centers have given similar findings. Our study was cross-sectional in design, and therefore potentially subject to recall bias; however, the data can be assumed to provide an estimate of the magnitude of the problem.

## Conclusion

Our results highlight the need to ensure that women of childbearing age and their health-care providers fully appreciate the importance of controlling asthma with appropriate medication use throughout pregnancy. Further studies should explore specific aspects of patients’ concerns and considerations about current asthma management during pregnancy, as well as the potential for implementation of relevant education programs.

## References

[1] The Global Initiative for Asthma (GINA); 2015. http://ginasthma.org/. Accessed Jan 2017.

[2] Moradi-Lakeh M, El Bcheraoui C, Daoud F, Tuffaha M, Kravitz H, Al Saeedi M (2015). Prevalence of asthma in Saudi adults: findings from a national household survey, 2013. BMC Pulm Med.

[3] Al-Moamary MS, Alhaider SA, Idrees MM, Al Ghobain MO, Zeitouni MO, Al-Harbi AS, Yousef AA, Al-Matar H, Alorainy HS, Al-Hajjaj MS (2016). The Saudi initiative for asthma - 2016 update: guidelines for the diagnosis and management of asthma in adults and children. Ann Thorac Med.

[4] Kwon HL, Triche EW, Belanger K, Bracken MB (2006). The epidemiology of asthma during pregnancy: prevalence, diagnosis, and symptoms. Immunol Allergy Clin N Am.

[5] National Heart L, Blood I, National Asthma E, Prevention Program A, Pregnancy Working G (2005). NAEPP expert panel report. Managing asthma during pregnancy: recommendations for pharmacologic treatment-2004 update. J Allergy Clin Immunol.

[6] Wise RA, Polito AJ, Krishnan V (2006). Respiratory physiologic changes in pregnancy. Immunol Allergy Clin N Am.

[7] Murphy VE, Namazy JA, Powell H, Schatz M, Chambers C, Attia J, Gibson PG (2011). A meta-analysis of adverse perinatal outcomes in women with asthma. BJOG Int J Obstet Gynaecol.

[8] Clark SL (1993). Asthma in pregnancy. National Asthma Education Program Working Group on asthma and pregnancy. National Institutes of Health, National Heart, lung and blood institute. Obstet Gynecol.

[9] Murphy VE, Gibson PG, Talbot PI, Kessell CG, Clifton VL (2005). Asthma self-management skills and the use of asthma education during pregnancy. Eur Respir J.

[10] Chambers K (2003). Asthma education and outcomes for women of childbearing age. Elsevier.

[11] Murphy VE, Namazy JA, Powell H (2011). A meta-analysis of adverse perinatal outcomes in women with asthma. BJOG.

[12] British Thoracic Society/Scottish Intercollegiate Guidelines Network, British Guideline on the Management of Asthma, Revised Edition. 2012.

[13] Enriquez (2006). Cessation of asthma medication in early pregnancy. Am J Obstet Gynecol.

